# Proof‐of‐principle of 3D‐printed track‐end detectors for dosimetry in proton therapy

**DOI:** 10.1002/mp.17515

**Published:** 2024-11-18

**Authors:** Vicky Bietenbeck, Claus Maximilian Bäcker, Jörg Wulff, Beate Timmermann, Christian Bäumer

**Affiliations:** ^1^ West German Proton Therapy Centre Essen Essen Germany; ^2^ West German Cancer Center (WTZ) University Hospital Essen Essen Germany; ^3^ Department of Particle Therapy University Hospital Essen Essen Germany; ^4^ German Cancer Consortium (DKTK) Essen Germany; ^5^ Department of Physics TU Dortmund University Dortmund Germany

**Keywords:** 3D printing, dosimetry, proton therapy

## Abstract

**Background:**

Dosimetric equipment in particle therapy (PT) is associated with high costs. There is a lack of versatile, tissue‐equivalent detectors suitable for in‐vivo dosimetry. Faraday‐cup (FC) type detectors are sensitive to stopped protons, that is, to track‐ends (TEs). They experience a renaissance in PT as they can cope with high dose rates. Owing to their simple functional principle, production of FC could benefit from the dynamic technological developments in additive manufacturing of sensors.

**Purpose:**

To build FC‐type detectors for PT by standard 3D‐printing. This study seeks to build an integrating, single‐channel (SC) FC for replacement of a traditional FC and a 2×2 array of FC elements indicating the feasibility of a spatially resolving detector.

**Methods:**

Samples of FCs were produced with a dual‐extruder 3D‐printer with polylactic‐acid filaments, which contained graphite in the conductive parts of the detector. Production was optimized in terms of materials and printing temperature. Samples were characterized by electrical tests and non‐destructive 3D x‐ray imaging. Beam tests were conducted at a clinical PT machine.

**Results:**

Operational FC‐type detectors for proton fields were printed. The detected charge of the SC FC corresponded qualitatively to the one of a traditional FC. A 2×2 FC array was fabricated in a single run. There was a linear relationship between the response of the individual FC elements and the machine output.

**Conclusions:**

3D‐printing is a viable method for producing low‐cost, tissue‐equivalent, FC‐type detectors for PT. They could potentially be used as TE detectors in anthropomorphic phantoms.

## 1 INTRODUCTION

There is renewed interest in Faraday‐cup (FC) detectors for the dosimetry in proton therapy (PT).^1^, ^2^ This trend is mainly caused by the inherent robustness of FCs against ultra‐high dose rates, which are for example, used in the frame of FLASH therapy concepts.^3^, ^4^, ^5^ By assessing the number of charged particles, FCs go beyond current dosimetry protocols, which standardize the measurement of absorbed dose. For short residual ranges, FCs indicate the number of track‐ends (TEs), which has been discussed as substitute quantity to drive the biology‐guided PT planning.^6^, ^7^, ^8^ Traditional FCs are bulky detectors, particularly those equipped with electric and magnetic suppressor fields. Due to the attached vacuum pump, their operation requires a rather high technical effort. Simplified FCs working in ambient conditions have been presented as an alternative^9^ and are commercially available.^1^ The current study seeks to build simplified FCs that can be tailored to a variety of dosimetric applications. The goal is to achieve this flexibility through the additive manufacturing of sensors, which has experienced quite some dynamics in the last few years.^10^, ^11^

## 2 MATERIALS AND METHODS

Compared to other type of sensors, the 3D‐printing of FCs is rather simple, because they consist of conducting parts embedded into insulating ones. The former ones were realized with filaments containing graphite (Protopasta, Vancouver, Washington, USA) with a diameter of 1.75 mm and the latter ones with standard polylactic‐acid (PLA) filaments. The fused deposition modeling was utilized, which heats, extrudes, and eventually deposits thermoplastic filaments onto one horizontal layer at a time to additively produce 3D structures.^12^ The Pro2 Plus 3D printer (RAISE3D, Irvine, USA) was employed together with the Slicer software IdeaMaker (RAISE3D, Irvine, USA). The fabrication parameters were optimized, particularly in terms of the printing temperature (195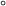, 210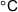, 220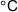 and 245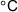). Two versions of FC‐type detectors with an increasing degree of structural complexity were manufactured. The first version concerned a modular, single‐channel (SC) FC with a height of 100 mm and a diameter of 30 mm. It was inspired by the first simplified FC for PT with metallic sensor,^9^ that is, it was designed to integrate the charge of a beamlet (spot) in lateral directions. The second version was a planar 2×2 array (body dimensions 25 mm × 25 mm ×17 mm) of FC elements with a height of 10 mm, a diameter of 5 mm and a pixel distance of 9 mm embedded in insulating PLA layers. While individual parts were printed separately and then assembled for the first version, the production of the FC array was accomplished in a single run, that is, by the alternating use of the two extruders.

PLA from Raise3D was used as non‐conductive printing material for the SC FC. A rectilinear grid filling pattern was used with a 90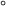 offset between the layers for the SC FC as well as for the ground and insulator of the array. The FC elements of the array were filled concentrically. A flow rate of 100%, a layer height of 0.2 mm and a print speed of 60 mm $s^{-1}$ were applied. The extruder diameter was 0.4 mm and the heating bed temperature was 60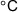. For the FC array, the printing parameters of both used materials were adapted to 208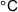 temperature, a 95% infill percentage, a flow rate of 90% and a print speed of 50 mm $s^{-1}$. A retraction of 0.5 mm and an 1.2 mm offset in *z*‐direction were set. After several tests, PLA from Tough Filament (Bambu Lab, Austin, Texas, USA) turned out to be the best choice as insulating printing material for the fine structures of the FC array. Tinned brass pins for breadboard circuits with a length of 8 mm and a diameter of 1 mm were pressed into holes of the 3D‐printed devices to facilitate the electrical connection of both types of FCs to a coaxial cable.

Electrical tests comprised short‐circuit tests with a common multimeter and dark current measurements with a Keithley Picoamperemeter 6487 (Keithley Instruments SRL, Cleveland, Ohio, USA). Non‐destructive imaging of the FC array was conducted with a NAEOTOM Alpha photon‐counting CT (Siemens, Forchheim, Germany) operated in ultra‐high resolution mode. Tests with proton fields were conducted in a gantry room of a ProteusPlus PT machine (IBA PT, Louvain‐la‐Neuve, Belgium), which was equipped with a universal nozzle and operated in the pencil‐beam scanning delivery mode. Individual central‐axis spots were applied on laterally centered FC detectors. The proton kinetic energy was 100 MeV. The linearity between the monitor units (MU)^13^ and the charge reading of the FC with a SuperMAX electrometer (Standard Imaging, Middelton, Wisconsin, USA) served as the main indication for the functioning of a proton beam detector. The water equivalent thickness (WET) of the used filament materials was determined by measuring the range pull‐back of a 150 MeV spot by 1 cm thick printed plates with a Giraffe multi‐layer ionization chamber (IBA Dosimetry, Schwarzenbruck, Germany).

Because the diameter of the SC FC does not capture the outer parts of a proton spot, a Monte‐Carlo simulation in RayStation 11B Ion PG (RaySearch Laboratories, Stockholm, Sweden) was conducted to assess the fraction of unregistered protons located in the outer lateral tail of a spot. In this research version of the treatment planning system, the TEs of primary and secondary protons, deuterons and alpha particles were scored. The geometrical efficiency $\eta_{30\mathrm{mm}}$ was estimated by the fraction of TEs in the volume of the FC and the TEs in the full simulated volume. This approach does not explicitly account for the negative charge contribution of residuals, which was approximated to be independent of lateral position.

## 3 RESULTS

**FIGURE 1:**
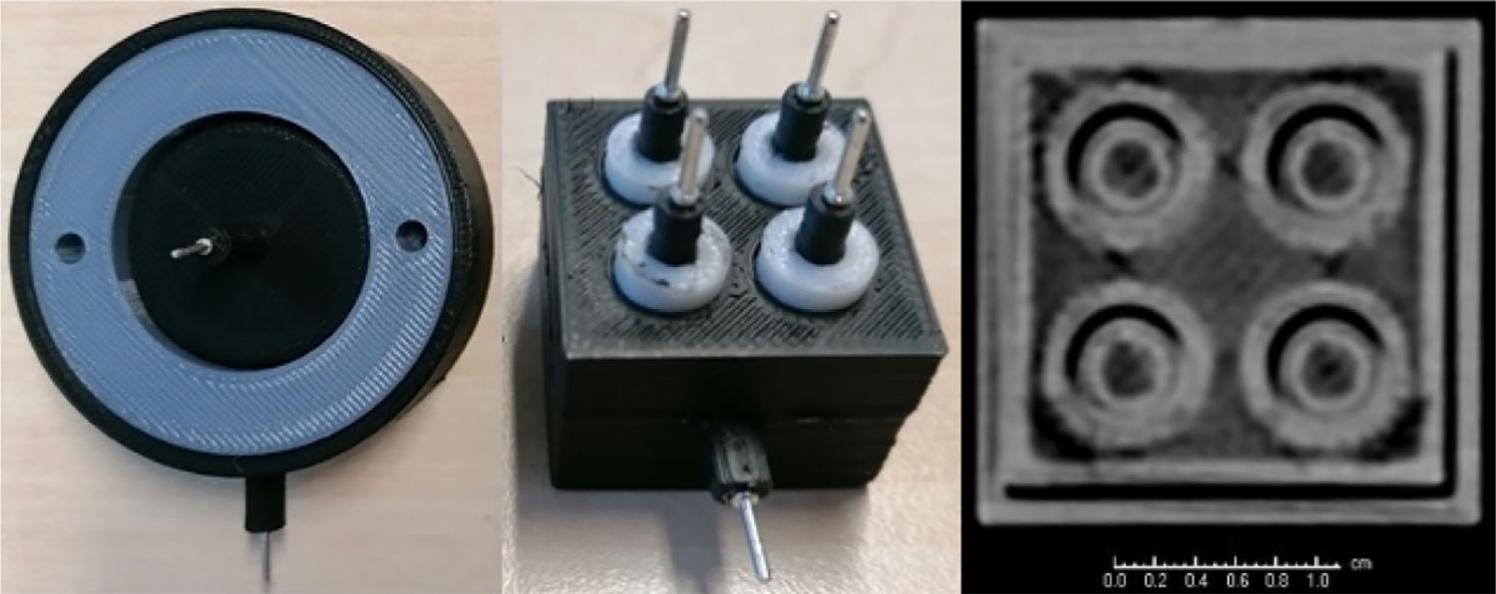
Photos of FC fabricated with additive manufacturing. Left: Photo of the modular large‐volume detector (30 mm/56 mm inner/outer diameter, 100 mm height). Middle/right: Photo/transversal x‐ray CT of the 2×2 array (25 mm/17 mm width/height of black cube). FC, Faraday‐cup.

At 195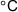, the 3D‐printed samples produced a poor surface above the support and structural defects in the top layers. The prints at 220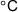 and 245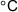 featured artefacts close to the side edges. Therefore, all FCs were fabricated with a temperature of 210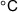. Photos of both versions of the 3D‐printed FC are shown in Figure 1 (left/middle). Computer‐aided design (CAD) files, CT slices and tables of measurement data are publicly available (10.5281/zenodo.12698838). The printing of the FC array had to be optimized to avoid electrical shortages. This concerns an increase of the fit size of the individual FC cylinders and an increase of the thickness of the insulator from 1  to 2 mm near the pins for electrical connection. Shortages within test samples during development could be correlated to misplaced conductive PLA using the high‐resolution CT image sets. Figure 1 (right) shows a CT slice of a fully functional FC array. An average dark current of 394 fA was measured for the SC FC and of 628 fA for the individual elements of the FC matrix. The measured average WET of the printed conductive plate was 1.02 cm (1.07 cm), for 95% (100%) infilll, i.e. the water‐equivalent ratio (WER) ranged between 1.02 and 1.07. The standard deviation of the WER in terms of printing reproducibility (spatial uniformity) of conductive PLA was ≈0.005 (≈0.008). The measured average WER for both types of used PLA was 1.05.

**FIGURE 2:**
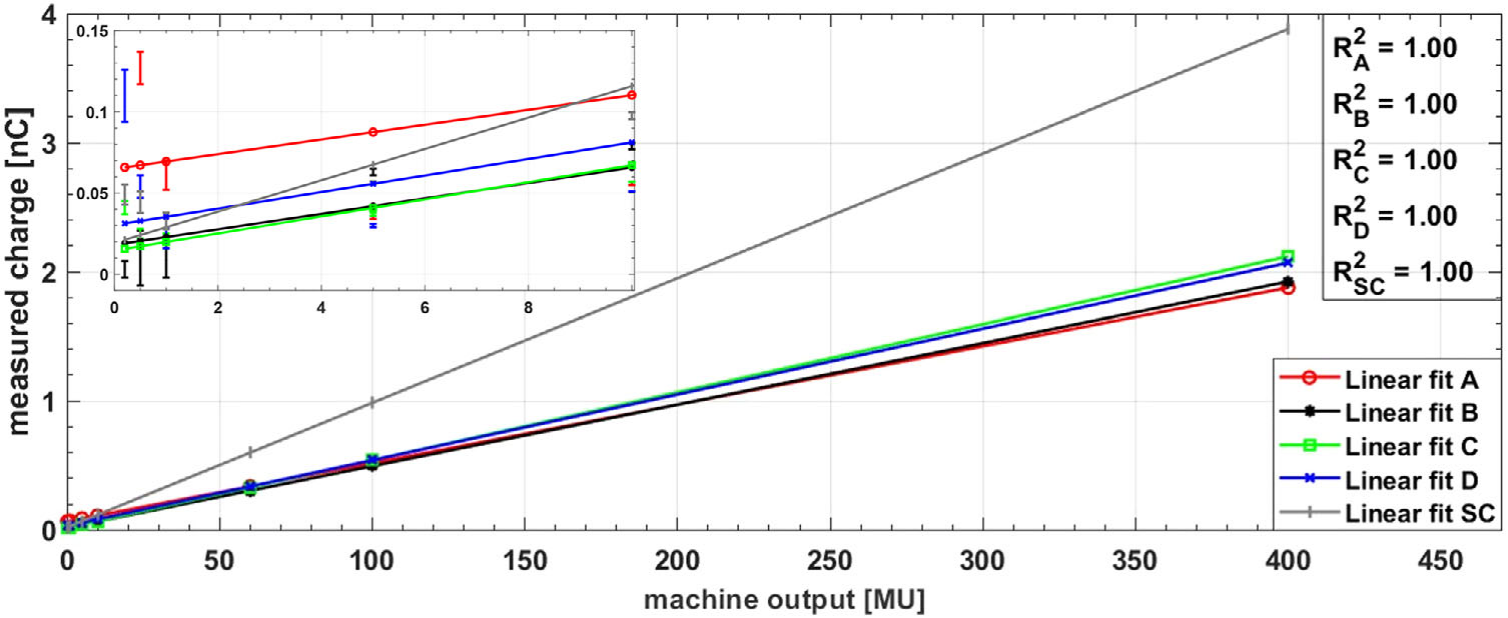
Test of the linearity of the detector response in terms of collected charge with machine output in terms of MU. SC FC and 2 × 2 array with pixels A‐D. FC, Faraday‐cup; SC, single‐channel.

Figure 2 shows the relationship between the charge measured with all detectors under test and the output of the proton machine. The linear detector response (coefficient of determination of the linear regressions ≈1.00) could be verified over two orders of magnitude. The non‐linear behaviour was more pronounced at low levels of MU. Here, it was typically on a level of a few percent but could be as high as about 30% for an individual pixel. The geometrical efficiency $\eta_{30\mathrm{mm}}$ was 70.5% according to the simulations. After correction by $\eta_{30\mathrm{mm}}$, the charge reading of the 3D‐printed, SC FC corresponded to 99.0% of the charge assessed with a traditional FC.^14^, ^15^

## 4 DISCUSSION

The dosimetric functionality of the 3D‐printed FCs was successfully demonstrated. A particularly conclusive test under beam conditions is the linear detector response with machine output (Figure 2). It needs to be noted that the lowest investigated machine output with 0.2 MU is a factor of ten higher than the minimum spot MU in a clinical treatment plan. On the other hand, the total plan may amount to more than 1000 MU. The nonlinearity at low MU deserves further investigation, particularly concerning an improved electronics readout. For a possible application in FLASH (Section 1), tests at ultra‐high dose rate, which was not available in our facility, should be performed.

The production of the 2 × 2 FC array was accomplished in a single run, that is, by the alternating use of the standard extruders, which represents a high technical level.^16^ This indicates the potential of building in‐house dosimetry equipment tailored to their need for PT centers. The used 3D printing technique is the lowest‐cost and most common one of this widespread tool.^11^, ^12^ While current trends for a democratization of PT refer to treatment machines and patient imaging/positioning,^17^ the high investment costs for specialized dosimetric devices have been neglected so far. Furthermore, FCs operate without bias voltage, which eases the path to in‐vivo dosimetry. The 2 × 2 FC array investigated in the current study indicates that this can be technically accomplished with multi‐channel FCs.^18^, ^19^ The integration as a TE detector in an anthropomorphic phantom, which could also be produced by additive manufacturing,^20^, ^21^, ^22^, ^23^ is a possible application in PT research. In this context, the advantage of PLA and graphite‐based filaments is their tissue‐equivalent density (WER = 1.02‐1.07). The uncertainty of the WER is quite low (<0.01). However, irregularities^23^ below the diameter of the probe spot (12.3 mm full‐width‐at‐half‐maximum) would not have been detected. The tissue equivalence for x‐ray imaging remains to be investigated in future studies.

## 5 CONCLUSIONS

3D‐printed FCs for dosimetry applications in proton therapy have been designed, built, and characterized in an exploratory project. The basic functionality as a radiation sensor was demonstrated, and potential paths to application in proton therapy were discussed.

## CONFLICT OF INTEREST STATEMENT

The authors declare no conflicts of interest.

## Data Availability

Source files and result tables are available at: 10.5281/zenodo.12698838.

## Footnotes

1 BC‐75 (Pyramid, Lexington, USA) and Qcup (Detector, Torino, Italy)
